# Mitochondrial fusion protein MFN2 interacts with the mitostatin-related protein MNS1 required for mouse sperm flagellar structure and function

**DOI:** 10.1186/2046-2530-3-5

**Published:** 2014-05-29

**Authors:** Melissa L Vadnais, Angel M Lin, George L Gerton

**Affiliations:** 1 Center for Research on Reproduction and Women’s Health, Department of Obstetrics and Gynecology, Perelman School of Medicine, University of Pennsylvania, 421 Curie Blvd., 1309 BRB II/III, Philadelphia, PA 19104-6160, USA

**Keywords:** MFN2, MNS1, Mitostatin, Sperm, Flagella, Axoneme, Mitochondria

## Abstract

**Background:**

Cilia and the sperm flagellum share many structural properties. Meiosis-specific nuclear structural 1 (MNS1) is a recently characterized protein that is abundantly expressed in post-meiotic spermatids and is required for proper flagellar and motile cilia formation. To explore the possible functions of MNS1, we performed a BLAST search and determined it is homologous to the conserved domain pfam13868, exemplified by mitostatin. This protein interacts with mitofusin 2 (MFN2), a protein that participates in regulating mitochondrial associations to subcellular organelles. We hypothesized that an association between MFN2 and MNS1 in the sperm is involved in flagellar biogenesis and function.

**Results:**

In the studies reported here, MFN2 was found in murine reproductive and somatic tissues high in ciliary content while MNS1 was present as two closely migrating bands in reproductive tissues. Interestingly, mitostatin was also present in reproductive tissues. Similar to *Mns1* and mitostatin, *Mfn2* was expressed in the testis as detected by RT-PCR. In addition, *Mfn2* and *Mns1* decreased in expression from pachytene spermatocytes to condensing spermatids as assessed by quantitative RT-PCR. Co-immunoprecipitation demonstrated an association between MFN2 and MNS1 in spermatogenic cells. Indirect immunofluorescence indicated that MFN2 and MNS1 co-localized to the sperm flagellum in freshly collected cauda epididymal sperm. MFN2 associated with the midpiece while MNS1 was present throughout the sperm tail in caput and cauda epididymal sperm. In spermatogenic cells, MFN2 was seen in the mitochondria, and MNS1 was present throughout the cell cytoplasm. MFN2 and MNS1 were present in detergent-resistant flagellar structures of the sperm.

**Conclusions:**

These results demonstrate that MFN2 and MNS1 are present in spermatogenic cells and are an integral part of the sperm flagellum, indicating they play a role in flagellar biogenesis and/or function.

## Background

**Figure 1:**
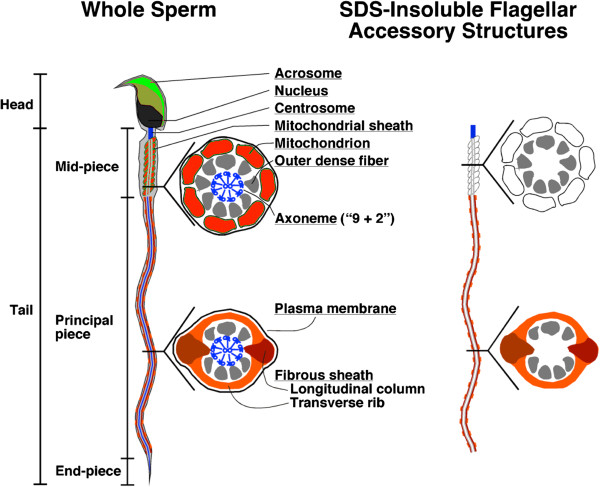
**Diagram of sperm cell.** Diagram detailing the major structural domains (midpiece, principal piece, and end piece) and key ultrastructural details (axoneme, ODFs, fibrous sheath, mitochondria, and annulus) of the sperm cell (Left). SDS-insoluble flagellar accessory structures of sperm tail (Right).

The sperm, a highly defined and compartmentalized cell, consists of the head (containing the nucleus and acrosomal vesicle) and the flagellum (Figure 1). The sperm flagellum is the specialized motile powerhouse of the cell and shares many similarities with cilia. The canonical ‘9 + 2’ microtubular axoneme runs down the center of the full length of the flagellum, which is delineated into three specialized structural compartments: the midpiece, the principal piece, and the end piece. The outer dense fibers (ODFs) are present throughout the midpiece and the principal piece. The midpiece is described by the presence of mitochondria wrapped in a spiral pattern around the nine ODFs that emanate from the outer doublets of the axoneme. The annulus is at the distal end of the midpiece. This is the demarcation point for the principal piece, which is characterized by the fibrous sheath. In this compartment, two longitudinal columns of the fibrous sheath replace ODFs 3 and 8 and substitute for the mitochondria in encapsulating the seven remaining ODFs. The end piece is found at the tip of the flagellum and contains no structural elements other than the axoneme.

A recently characterized flagellar protein, meiosis-specific nuclear structural 1 (MNS1), is abundantly expressed in post-meiotic spermatids and is required for proper flagellar formation and function [1]. Male mice deficient in MNS1 display decreased sperm production and are sterile because their sperm are immotile. The microtubules and ODFs of the flagella are disrupted, resulting in abnormal, short sperm tails. Similarly, the motile cilia of the trachea of MNS1-null males exhibit defects in the outer dynein arms of their axonemes. Based upon the extensive homology to proteins that comprise axonemal structures in a wide variety of organisms from *Chlamydomonas* to mammals, we postulate that mutations in MNS1 or associated proteins contribute to male infertility and/or ciliopathies in humans.

To elucidate a mechanism involving MNS1 and to discover candidates for associating proteins, we performed a BLAST search of the Conserved Domain Database [2]. We identified a domain structure, pfam13868, which is characterized by mitostatin (also referred to as trichoplein and keratin filament binding protein), a protein that prevents cilia formation during the cell cycle and promotes their assembly during quiescence [3-5]. In somatic cells, outer dense fiber protein 2 (ODF2) recruits mitostatin, which, in turn, enlists ninein. In addition, mitostatin is involved in regulating mitochondrial-ER binding via mitofusin 2 (MFN2) [6]. In mitochondria, MFN2 is an outer membrane protein that participates in the fusion, maintenance, and operation of the mitochondrial network [7]. Although not well described in sperm, MFN2 has been localized to the flagellum in boar sperm [8,9].

While MNS1 is critical for male fertility, its physiological functions and molecular mechanisms of interactions remain unknown. From the structural similarity of MNS1 to mitostatin, we can envision how this protein may function during spermatogenesis and in sperm. We hypothesize that MNS1 participates in a complex with MFN2 and ODF2 to anchor the mitochondria to the midpiece. In this investigation, we show that MFN2 is present in spermatogenic cells and epididymal sperm and, like MNS1, associates with the sperm flagellum. These studies are the first to report that MNS1 co-immunoprecipitated with MFN2 and that these proteins co-localized to the sperm flagellum.

## Methods

### Spermatogenic cell isolation

All animal procedures were approved by the University of Pennsylvania Institutional Animal Care and Use Committee and ethical guidelines were in accordance with the National Research Council’s publication *Guide for Care and Use of Laboratory Animals* and the *Guide for the Care and Use of Agricultural Animals in Agricultural Research and Teaching*, Federation of Animal Science Societies, First Revised Edition, 1999. Mixed germ cells were prepared from decapsulated testes of adult male mice (C57BL/6 retired breeders; Charles River Laboratories, Wilmington, MA, USA) by sequential dissociation with collagenase and trypsin-DNase I [10]. In some cases, spermatogenic cells were isolated from ‘Red-Green’ mice (B6D2F1-Tg(CAG/su9-DsRed2, Acr3-EGFP)RBGS002Osb) which express green fluorescent protein (GFP) in the acrosome precursors of spermatogenic cell and red fluorescent protein (RFP) in mitochondria of all cells [11]. To purify populations of pachytene spermatocytes, round spermatids, and condensing spermatids, the mixed germ cells were separated at unit gravity in a gradient of 2% to 4% bovine serum albumin (BSA) in Eagle’s Essential Medium with Earle’s Salts [12,13]. Both the pachytene spermatocyte and round spermatid populations were at least 85% pure as determined by microscopic examination and differential counting with a hemocytometer; whereas, the condensing spermatid population was approximately 40% to 50% pure, with the balance primarily being anucleate residual bodies and round spermatids.

### Purification of caput and cauda epididymal sperm

Sperm were collected from the caput and cauda epididymides of male mice (C57BL/6 retired breeders; Charles River Laboratories) by cutting the epididymides and extruding the sperm at 37°C into phosphate-buffered saline (PBS: 2.68 mM KCl, 136.09 mM NaCl, 1.47 mM KH_2_PO_4_, 8.07 mM Na_2_HPO_4_, pH 7.4 containing protease inhibitors (Roche, San Francisco, CA, USA)). Caput epididymal sperm were purified by centrifugation at 400 × *g* for 20 min at room temperature through a 35% PureSperm 100 solution (MidAtlantic Diagnostics, Mt. Laurel, NJ, USA) in PBS. Purified sperm were collected from the pellet, resuspended in PBS at 4°C, counted, and assessed for purity. Cauda epididymal sperm were collected by centrifugation at 800 × *g* for 5 min at room temperature, resuspended in PBS at 4°C, and counted.

### Incubation of sperm in epididymis-like, non-capacitating, or capacitating conditions

Cauda epididymal sperm were collected and incubated in a physiological solution that mimicked epididymal luminal fluid [14] or were collected and incubated under non-capacitating or capacitating conditions [15]. To simulate the epididymal lumen environment, sperm were allowed to swim out from the caudae epididymides into 2 mL of physiological solution that mimicked epididymal luminal fluid (50 mM NaCl, 50 mM potassium gluconate, 1.2 mM MgSO_4_, 0.6 mM CaCl_2_, 4 mM sodium acetate, 1 mM trisodium citrate, 6.4 mM NaH_2_PO_4_, and 3.6 mM Na_2_HPO_4_ then adjusted to pH 6.8 and approximately 350 to 360 mOsmol/kg H_2_O with raffinose at 37°C. For non-capacitating or capacitating conditions, sperm were allowed to swim out from the caudae epididymides into 2 mL modified Whitten medium (MW: 15 mM HEPES, 1.2 mM MgCl_2_, 100 mM NaCl, 4.7 mM KCl, 1 mM pyruvic acid, 4.8 mM lactic acid hemi-calcium salt, 5.5 mM glucose, pH 7.35) at 37°C. Epididymal tissue was removed, and the sperm were washed at 100 × *g* for 1 min in a clinical centrifuge to remove any gross tissue debris. The sperm were resuspended in a final volume of 5 mL of either physiological solution mimicking epididymal uminal fluid or MW then centrifuged at 500 × *g* for 8 min in a round-bottomed tube. Sperm from the resultant pellet were counted, assessed for motility, and diluted for use. In all cases, large-bore plastic transfer pipettes or large-orifice pipette tips were used to minimize damage to the sperm membranes. After collection and washing, sperm were incubated under physiological conditions in medium mimicking epididymal luminal fluid, non-capacitating conditions in MW, or capacitating conditions in MMW (MW with 10 mM NaHCO_3_, 3 mM 2-hydroxypropyl-β-cyclodextrin) for 1 h in a 37°C water bath at a final concentration of 4 × 10^6^ sperm in 600 μL. After incubation, sperm were processed for indirect immunofluorescence as described below.

### Purification of SDS-insoluble sperm heads and tails

Cauda epididymal sperm were homogenized in 1% sodium dodecyl sulfate (SDS), 75 mM NaCl, 24 mM EDTA, protease inhibitors, pH 6.0 (S-EDTA), layered onto a 1.6 M sucrose cushion in S-EDTA and centrifuged at 5000 × *g* for 1 h at room temperature [16]. The SDS-resistant head structures (nuclei) were collected from the pellet. The SDS-resistant tail structures (flagellar accessory structures lacking the plasma membrane and axonemes) were collected from the interface (Figure 1). The purity was visually assessed by light microscopy to determine adequate head and flagellar accessory structure (‘tail’) separation.

### Detergent fractionation of spermatogenic and sperm cells

Mixed germ cells and cauda epididymal sperm were collected as described above. For each cell population, 10^6^ cells in 100 μL of PBS containing 0.1% Triton X-100 were incubated at room temperature for 10 min then centrifuged at 10,000 × *g* for 1 min. Triton X-100 soluble and insoluble proteins were collected from the supernatant and pellet, respectively. Further detergent extraction was performed on the Triton X-100 insoluble proteins (pellets). One hundred microliters of S-EDTA was added to the Triton X-100 insoluble protein pellet, incubated at room temperature for 10 min, homogenized, and centrifuged at 10,000 × *g* for 1 min. The Triton X-100/S-EDTA soluble and insoluble proteins were collected from the supernatant and pellet, respectively.

### Protein extraction and immunoblot analysis

The spermatogenic cells and sperm cells were concentrated by centrifugation, washed in 1 mL of PBS, resuspended in sample buffer (62.5 mM Tris-HCl, pH 6.8, 1.67% SDS, 10% glycerol), and boiled for 5 min. Detergent extracted proteins were mixed with sample buffer and boiled for 5 min. Other tissues were homogenized, sonicated, and boiled in the sample buffer. After centrifugation, the supernatants were recovered and saved. Protein concentrations were determined by the BCA protein assay (Pierce Chemical, Rockford, IL, USA) and then dithiothreitol (DTT) and bromophenol blue were added to final concentrations of 100 mM and 0.002%, respectively. The samples were boiled for 5 min, and protein samples (15 μg per lane) were separated by SDS-polyacrylamide gel electrophoresis (PAGE) in 10% polyacrylamide gels [17]. The gels were then transferred to polyvinylidene difluoride membranes [18]. After the membranes were blocked with TBSS (500 mM NaCl, 25 mM Tris-HCl, pH 8.0) or TBST (125 mM NaCl, 25 mM Tris-HCl, pH 8.0, 0.1% Tween 20) containing 5% BSA, they were incubated with primary antibody (1:500 anti-MNS1, 1:100 anti-trichoplein (Santa Cruz Biotechnology, Dallas, TX, USA) or 1:1,000 anti-MFN2 (AbCam, Cambridge, MA, USA)) for 1 h. The antibody against MNS1 has been previously characterized [1]. We affinity-purified the MNS1 antibody from anti-MNS1 sera using the immunoblot purification method [19]. After washing with TBSS or TBST, the blots were incubated for 1 h with secondary antibody (donkey anti-rabbit IgG or anti-mouse IgG conjugated with horseradish peroxidase (GE Healthcare, Milwaukee, WI, USA), 1:5,000 in 5% BSA in TBSS or TBST) and, after washing with TBSS or TBST, the bound enzyme was developed with the ECL kit (GE Healthcare) according to the manufacturer’s directions.

### Co-immunoprecipitation of spermatogenic cells

Co-immunoprecipitation was performed using the Direct Magnetic IP/Co-IP Kit (Pierce Chemical) according to manufacturer’s instructions. Briefly, mixed germ cells, collected as described above, were lysed with ice-cold IP Lysis/Wash Buffer on ice for 5 min. After centrifugation at 13,000 × *g*, the protein concentration was determined by the BCA protein assay (Pierce Chemical). Five micrograms of MNS1 purified antibody or MFN2 antibody was coupled to the N-hydroxysuccinimide-activated magnetic beads. Two milligrams of isolated mixed germ cell protein was added to the antibody-coupled magnetic beads and incubated with rotation for 2 h. After washing the unbound protein from the beads, the bound protein was eluted from the beads with Elution Buffer. Unbound antibody, unbound antigen, and elution were analyzed by SDS-PAGE and immunoblotting as described above. Blots were probed with anti-MNS1 and anti-MFN2 antibodies.

### Indirect immunofluorescence analysis

One million mixed germ cells from Red-Green mice were collected as described above and attached to polylysine-coated coverslips for 15 min. Cells were fixed in 0.1 M PIPES, pH 6.8, containing 2 mM EGTA, 20 mM MgCl_2_, 2% paraformaldehyde, and 0.1% glutaraldehyde. After washing with 0.15 M Tris-HCl, pH 7.4, the cells were permeabilized for 2 min with 0.2% Triton X-100 in PBS [12]. In other experiments, one million caput or cauda epididymal sperm, collected as described above, were attached to polylysine-coated coverslips for 15 min then fixed and permeabilized with ice-cold acetone/methanol (1:1) for 1 min.

After extensive washing with PBS, the coverslips were incubated at 37°C for 1 h with 10% goat serum in PBS (blocking solution). The coverslips were incubated overnight at 4°C with primary antibodies (1:100 anti-MNS1 and/or 1:200 anti-MFN2) diluted in blocking solution. The following day, the coverslips were incubated for 1 h at 37°C with the corresponding Alexa Fluor 488-conjugated secondary antibodies (1:500; GE Healthcare) diluted in blocking solution. In cases of dual staining, coverslips were incubated for 1 h at 37°C with anti-rabbit Alexa Fluor 488- and anti-mouse Alexa Fluor 568-conjugated secondary antibodies (1:500; GE Healthcare) diluted in blocking solution. Finally, the coverslips were mounted on slides using 15 μL of Fluoromount-G (Southern Biotechnology Associates, Birmingham, AL, USA), observed with a Nikon Eclipse TE 2000-U inverted microscope (Nikon Instruments, Melville, NY, USA), and photographed with a CFW-1610C digital FireWire camera (Scion, Frederick, MD, USA) using the NIH ImageJ imaging software available online (http://rsb.info.nih.gov/ij/). Nomarski differential interference contrast micrographs were photographed in parallel with the fluorescence images. The polarizing elements in the differential interference contrast light path were removed prior to capturing the images. Negative controls using pre-immune sera, normal sera, or secondary antibody alone were also used to check for specificity.

### RNA preparation and RT-PCR

RNA was prepared from spermatogenic cells, testes, and various somatic tissues using TRI Reagent (Sigma-Aldrich Corp., St. Louis, MO, USA). Reverse transcription using 1 μg mRNA was performed using SuperScript II Reverse Transcriptase according to the manufacturer’s instructions (Invitrogen Corp., Carlsbad, CA, USA). Products were amplified with 25 cycles using the appropriate primers (Table 1) with Ex Taq DNA polymerase (Takara Co., Tokyo, Japan).

**Table 1:** List of primer sets used for collection of experimental data

**Primer name**	**Application**	**Forward primer (5’-3’)**	**Reverse primer (5’-3’)**
*Mfn2*	RT-PCR	GCC AGC TTC CTT GAA GAC AC	GCA GAA CTT TGT CCC AGA GC
*Mns1*	RT-PCR	CAG TTC CTC GCA GAC AAA CA	GGC CTT GCT ATG AAG ACT CG
*Mitostatin*	RT-PCR	GAC AAA GGC AGA GGC TTG AC	CAA GCC ATA ACA ACC CGA CT
*Gapdh*	RT-PCR	GGT GAA GGT CGG TGT GAA CG	CTC GCT CCT GGA AGA TGG TG
*Mfn2*-qPCR1 1	qRT-PCR	ATG TTA CCA CGG AGC TGG AC	AAC TGC TTC TCC GTC TGC AT
*Mfn2*-qPCR2 2	qRT-PCR	CTA TGA GCG ACT GAC CTG GA	CAC TGG CGT ATT CCA CAA AC
*Mns1*-qPCR1 1	qRT-PCR	CCT CAA AGA ACA TGC TGC AA	GGC CTT GCT ATG AAG ACT CG
*Mns1*-qPCR2 2	qRT-PCR	AAG CAG CTG GAG GAA ACA CT	TTG ATG ATC TCT GCC TGC TC
*Gapdh*-qPCR	qRT-PCR	AAC TTT GGC ATT GTG GAA GG	GGA TGC AGG GAT GAT GTT CT
*Ribosomal Protein S16*	qRT-PCR	AGA TGA TCG AGC CGC GC	GCT ACC AGG GCC TTT GAG ATC GA

### Preparation of RNA and quantitative RT-PCR

For quantitative RT-PCR assays, products were amplified with the SYBR Green PCR Master Mix (Applied Biosystems, Carlsbad, CA, USA) and analyzed with the ABI 7900 HT Sequence Detection system (Applied Biosystems). The following PCR protocol was used: (1) denaturation at 95°C for 1 s; (2) amplification and quantification at 60°C for 20 s then repeated for 40 cycles; (3) a dissociation curve program at 95°C for 15 s, 60°C for 15 s, 95°C for 15 s; (4) cooling at 4°C. Amplicons were analyzed by generating a dissociation curve and determining the threshold cycle (Ct) value for each transcript. The relative quantification of gene expression was analyzed by the 2(-Delta Delta C(T)) method [20]. The mRNAs corresponding to ribosomal protein S16 (accession number: BC082286) and GAPDH (accession number: M32599) were used as controls [21]. Primer sets used are listed in Table 1.

## Results

### MNS1 shares a conserved region with mitostatin

**Figure 2:**
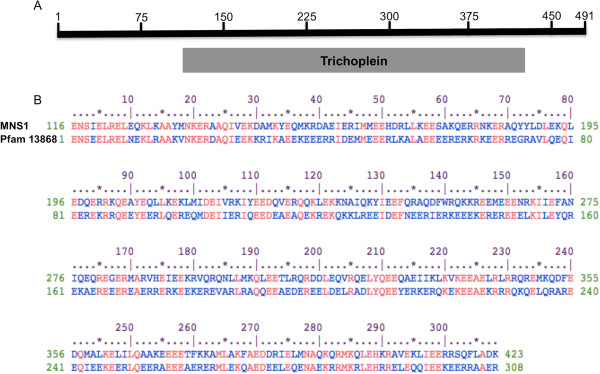
***In silico *** **analysis of the protein sequence of MNS1.** BLAST analysis of the protein MNS1 demonstrated that it shares a conserved domain known as the mitostatin region (pfam13868). **(A)** The mitostatin region aligns with amino acids 116 to 423 of MNS1. **(B)** In this area, there is a 40% similarity in of the MNS1 amino acid sequence with consensus sequence for the mitostatin domain. http://www.ncbi.nlm.nih.gov/Structure/cdd/wrpsb.cgi?SEQUENCE=NP_032639.1&FULL.

We performed *in silico* analysis of the protein sequence of MNS1 to see if there were any regions with recognizable conserved stretches that might indicate a possible function. The 491-amino acid sequence (accession number: NP032639) for MNS1 was used in a BLAST search to identify homologous sequences or shared conserved domains. Analysis of the Conserved Domain Database identified the mitostatin region (pfam13868) as a significant ‘hit’. MNS1 and the consensus sequence for pfam13868 shared a 40% amino acid homology (Figure 2). Within the 308-amino acid conserved domain match between MNS1 and pfam13868, there were multiple identical or similar amino acid residues indicating a structural relationship between these two sequences. These findings indicate that MNS1 may function similar to mitostatin.

### *Mfn2* is expressed in the testis

**Figure 3:**
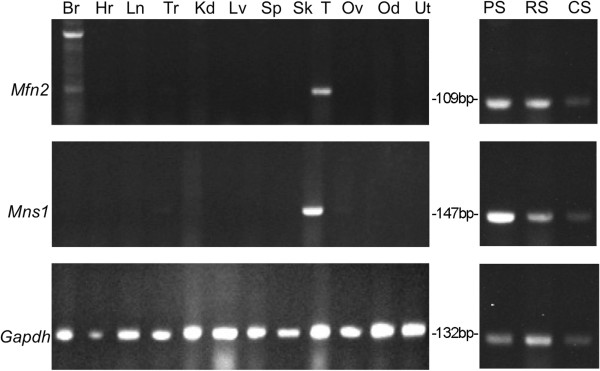
**Expression of *Mfn2*, *Mns1*, and *Gapdh *** **in somatic and reproductive tissues as detected by RT-PCR. ***Mfn2* was transcribed in brain and testis while *Mns1* was expressed in the testis. Both genes were expressed in spermatogenic cells. Br: brain, Hr: heart, Ln: lung, Tr: trachea, Kd: kidney, Lv: liver, Sp: spleen, Sk: skeletal muscle, T: testis, Ov: ovary, Od: oviduct, Ut: uterus, PS: pachytene spermatocytes, RS: round spermatids, CS: condensing spermatids. Amplicon size for *Mfn2*, *Mns1*, *Gapdh* was 109, 147, and 132 base pairs (bp), respectively.

By analogy with mitostatin, which is involved in ciliogenesis [3-5] and in regulating mitochondrial-ER binding via MFN2 [6], we hypothesized that MNS1 may interact with MFN2. RT-PCR was used to determine the expression of *Mfn2*, *Mns1*, and mitostatin in somatic tissues, reproductive tissues, and spermatogenic cells. Mitostatin was present in kidney and testis (data not shown). *Mfn2* mRNA was found in brain, testis, and, faintly, in trachea. *Mns1* mRNA was detected in testis and faintly in trachea and oviduct. Both genes were expressed in spermatogenic cells (Figure 3). Qualitatively, the levels of each mRNA found in pachytene spermatocytes appeared much higher than the amounts observed in round spermatids. Both genes were expressed in similar tissues lending further support to the hypothesis that they may interact. *Gapdh* was used as a loading control.

### *Mfn2* mRNA levels decrease from pachytene spermatocytes to condensing spermatids

**Figure 4:**
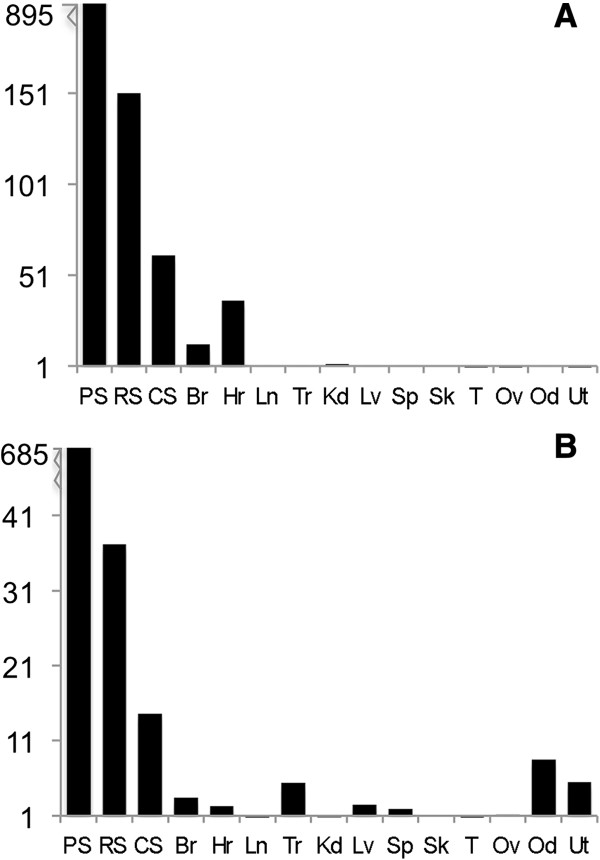
**Quantitative RT-PCR of *Mfn2 *** **(A) and *Mns1 *** **(B) in somatic and reproductive tissues.** The expression level was determined as the fold-increase over baseline in mouse skeletal muscle. *Gapdh* and ribosomal protein S*16* were used as expression control genes. *Mfn2* and *Mns1* were highly expressed in spermatogenic cells, decreasing from pachytene spermatocytes to condensing spermatids. Br: brain, Hr: heart, Ln: lung, Tr: trachea, Kd: kidney, Lv: liver, Sp: spleen, Sk: skeletal muscle, T: testis, Ov: ovary, Od: oviduct, Ut: uterus, PS: pachytene spermatocytes, RS: round spermatids, CS: condensing spermatids. Numbers on the ordinate represent fold-increase over baseline (mouse skeletal muscle).

Quantitative RT-PCR of mRNAs from purified populations of mouse spermatogenic cells and somatic tissues were used to explore the levels of *Mfn2* and *Mns1*. Both genes were highly expressed in spermatogenic cells, decreasing from pachytene spermatocytes to condensing spermatids (Figure 4). The *Mfn2* gene was also expressed in brain and heart. *Mns1* was transcribed in brain, heart, trachea, liver, spleen, oviduct, and uterus. For both mRNAs, the levels present in pachytene spermatocytes were substantially higher than those found in round spermatids and condensing spermatids. *Mfn2* scored at 894 relative units for pachytene spermatocytes compared to the 151 units for round spermatids. In addition, *Mns1* in pachytene spermatocytes was measured at 685 relative units compared to the 37 units found for round spermatids. On the other hand, the levels for these mRNAs in somatic tissues never exceeded 5% of the pachytene spermatocyte values. Two primer sets were used to confirm quantitative RT-PCR results for *Mfn2* and *Mns1* (Table 1). Neither primer set was able to recognize gene expression in the testis; however, when each primer set was used in RT-PCR, *Mfn2* mRNA was found in spermatogenic cells, brain, testis, and trachea. *Mns1* mRNA was detected in spermatogenic cells, testis, trachea, and oviduct (data not shown). These results are similar to what was found with the primer sets used in RT-PCR (data shown in Figure 3). The reason for the inability of these primers to work with RNA from whole testis when they worked with RNA from isolated populations of germ cells is unknown at present. Although the quantitative RT-PCR results for testis were enigmatic, these data demonstrate that *Mfn2* and *Mns1* are highly expressed in spermatogenic cells just prior to and during the period of differentiation when the biogenesis of the flagellum occurs.

### MFN2 is found in reproductive and somatic tissues with a high ciliary content

**Figure 5:**
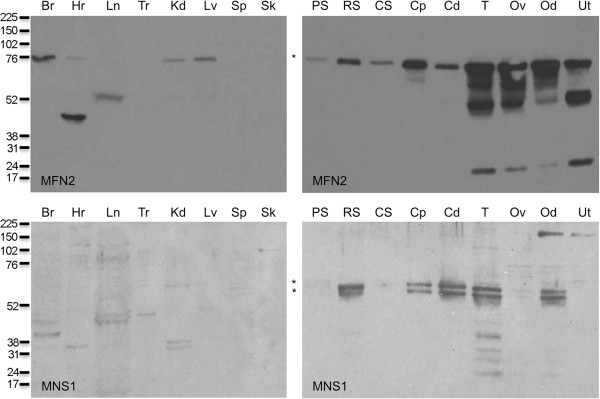
**Immunoblot of somatic and reproductive tissues probed with antibodies against MFN2 (top) or MNS1 (bottom).** MFN2 was seen in brain, heart, kidney, and liver (top, left panel) and was found in all reproductive tissues examined (top, right panel). MNS1 was seen faintly in the kidney (bottom, left panel) and was present as two closely migrating bands in all reproductive tissues and isolated cells examined except pachytene spermatocytes, condensing spermatids, and uterus (bottom, right panel). Br: brain, Hr: heart, Ln: lung, Tr: trachea, Kd: kidney, Lv: liver, Sp: spleen, Sk: skeletal muscle, PS: pachytene spermatocytes, RS: round spermatids, CS: condensing spermatids, Cp: caput epididymal sperm, Cd: epididymal sperm, T: testis, Ov: ovary, Od: oviduct, Ut: uterus. Numbers to the left of the panels indicate the molecular weights of standard proteins (×10^-3^). Asterisk (*) indicates expected size (MFN2: *M*_*r*_ of 80 and MNS1: *M*_*r*_ of 60) of protein.

Using SDS-PAGE and immunoblotting, proteins from somatic tissues, reproductive tissues, spermatogenic cells, caput epididymal sperm, and cauda epididymal sperm were probed with antibodies against MFN2 and MNS1 (Figure 5). MFN2 was seen in tissues from brain, kidney, liver, and, faintly, heart. This protein was found in all reproductive tissues, spermatogenic cells, and epididymal sperm examined. Three additional immunoreactive bands (*M*_*r*_ of 70,000, 52,000, and 17,000) were noted only in the testis, ovary, oviduct, and uterus samples.

MNS1 was seen as a faint, single band in tissue from kidney and as two closely migrating bands (*M*_*r*_ of 71,600 and 63,800) in reproductive tissues, being found in all tissues examined except pachytene spermatocytes, condensing spermatids, and uterus (Figure 5: Bottom). Four bands migrating at *M*_*r*_ of 43,200, 38,400, 33,200, and 29,200 were present only in the testis. Two bands migrating at approximately *M*_*r*_ of 37,000 have been reported [1]. Note that MNS1 appears to be greatly reduced in condensing spermatids relative to round spermatids and epididymal sperm; this likely results from the fact that the condensing spermatids lose their flagella and cytoplastic precursors of residual bodies as a consequence of the proteolytic enzymes used to dissociate the seminiferous tubules into single cell suspensions. In these experiments, we show that MFN2 and MNS1 were both present in spermatogenic cells and epididymal sperm. We also confirmed the presence of mitostatin in spermatogenic cells and reproductive tissues (data not shown).

### MFN2 and MNS1 form a protein complex in spermatogenic cells

**Figure 6:**
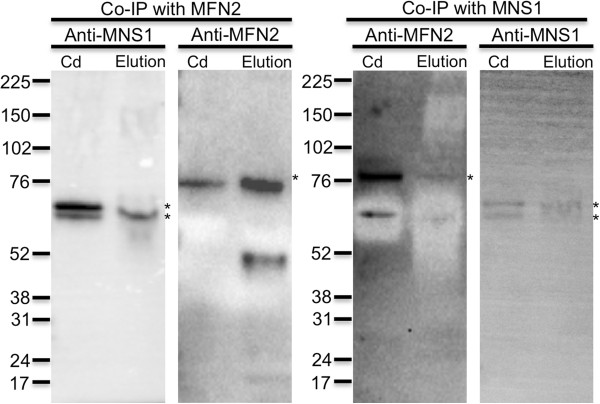
**Co-immunoprecipitation of protein from spermatogenic cells with MFN2 (left panel) or MNS1 (right panel).** The left panel displays the co-immunoprecipitates with MFN2 probed with antibodies against MNS1 or MFN2. MNS1 co-immunoprecipitated with MFN2 (left panel). The right panel displays the co-immunoprecipitates with MNS1 probed with antibodies against MFN2 or MNS1. MFN2 co-immunoprecipitated with MNS1 (right panel). Cauda epididymal sperm (Cd) was included on each membrane as a control. Numbers at the left of the panels indicate the molecular weights of standard proteins (×10^-3^). Asterisk (*) indicates expected size (MFN2: *M*_*r*_ of 80 and MNS1: *M*_*r*_ of 60) of protein.

Co-immunoprecipitation experiments were performed to determine if MFN2 associates with MNS1 in murine spermatogenic cells. MNS1 co-immunoprecipitated with MFN2 (Figure 6: Left). Similar to what was seen in reproductive tissues, spermatogenic cells, and epididymal sperm (Figure 5: Bottom), MNS1 was present as two closely migrating bands although the top band was extremely faint. Furthermore, MFN2 co-immunoprecipitated with MNS1 (Figure 6: Right). Cauda epididymal sperm were included on each membrane as a control. This set of results demonstrated that MFN2 and MNS1 formed a protein complex in spermatogenic cells.

### MFN2 immunofluorescence pattern changes from the midpiece to the entire flagellum after incubation

**Figure 7:**
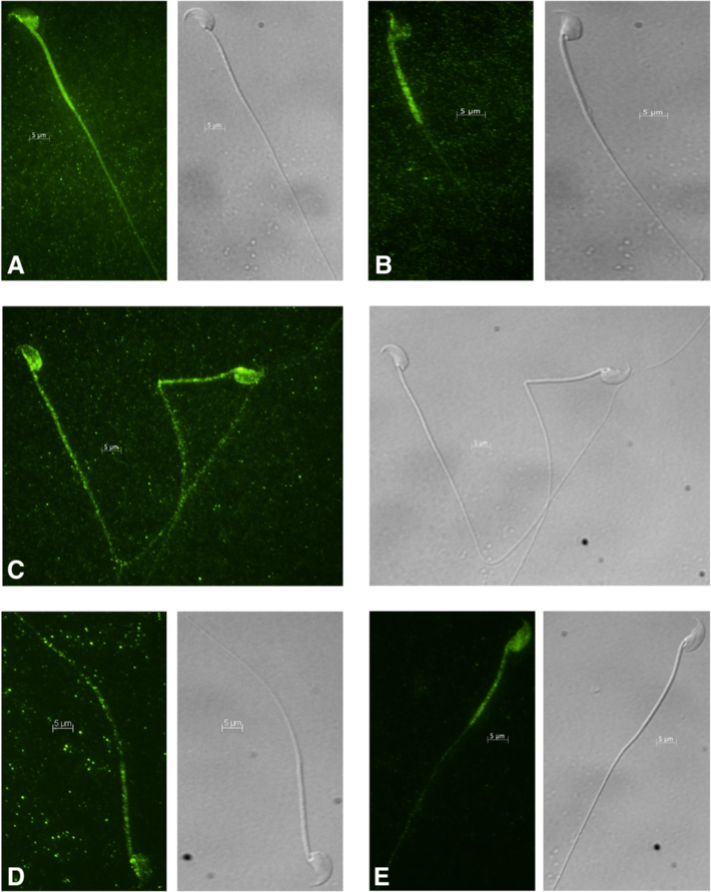
**Immunofluorescence of sperm probed against anti-MFN2.** Immunofluorescence using freshly collected caput epididymal sperm **(A)**, freshly collected cauda epididymal sperm **(B)**, cauda epididymal sperm incubated in non-capacitating medium **(C)**, cauda epididymal sperm incubated in capacitating medium **(D)**, or cauda epididymal sperm incubated in medium mimicking epididymal luminal fluid **(E)** and probed against anti-MFN2 was performed. MFN2 was localized to the sperm midpiece and post-acrosomal region in caput epididymal sperm, cauda epididymal sperm, and in sperm maintained in medium mimicking epididymal luminal fluid. In sperm incubated in non-capacitating or capacitating media the fluorescence was detected in the post-acrosomal region and throughout the flagellum. A 5 μm bar is indicated on all images. Nomarski differential interference contrast micrographs are located to the right of each fluorescent image.

Indirect immunofluorescence was used to examine the localization of MFN2 in caput epididymal sperm and cauda epididymal sperm. Immediately after collection, MFN2 was localized to the midpiece and post-acrosomal regions of sperm from the caput and cauda regions of the epididymis (Figure 7A and B). Similar results were seen when cauda epididymal sperm were incubated in low pH (pH 6.8), high osmolality conditions in medium mimicking epididymal luminal fluid (Figure 7C). However, when sperm were incubated in conditions standardly used to handle mouse sperm, that is, either non-capacitating or capacitating media (pH 7.35), the localization pattern observed by immunofluorescence changed in greater than 80% of the sperm population (Figure 7D and E). Fluorescence continued to be present in the post-acrosomal region of the sperm head but the MFN2 staining appeared throughout the sperm tail.

### MNS1 localizes to the sperm flagellum

**Figure 8:**
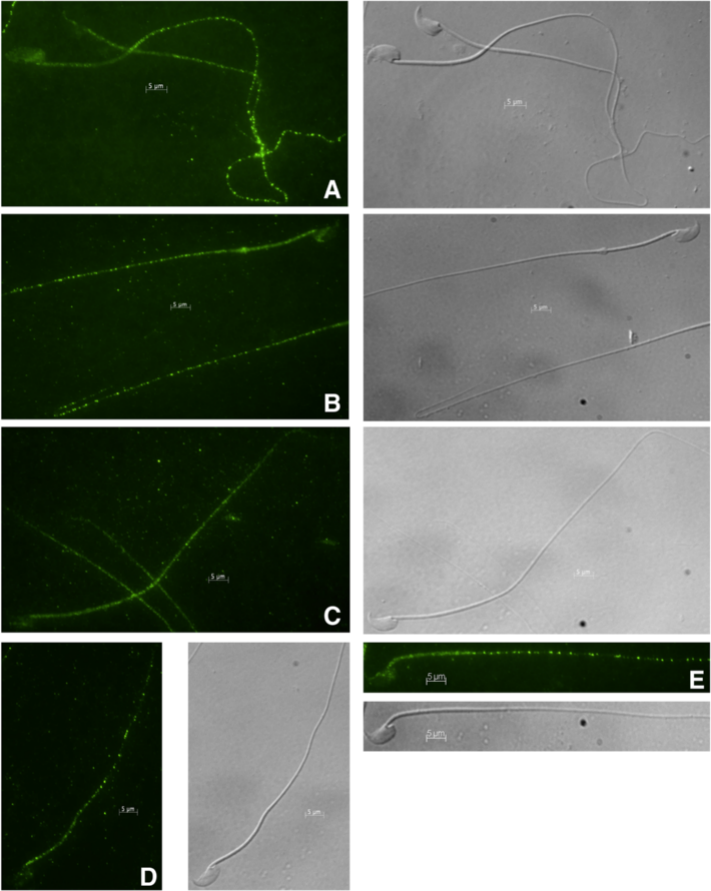
**Immunofluorescence of sperm probed against anti-MNS1.** Immunofluorescence using freshly collected caput epididymal sperm **(A)**, freshly collected cauda epididymal sperm **(B)**, cauda epididymal sperm incubated in non-capacitating medium **(C)**, cauda epididymal sperm incubated in capacitating medium **(D)**, or cauda epididymal sperm incubated in medium mimicking epididymal luminal fluid **(E)** and probed against anti-MNS1 was performed. MNS1 localized to the sperm flagellum and post-acrosomal region. A 5 μm bar is indicated on all images. Nomarski differential interference contrast micrographs are located to the right of each fluorescent image.

The localization of MNS1 in caput epididymal sperm and cauda epididymal sperm was determined using indirect immunofluorescence. MNS1 was detected as punctate staining throughout the length of the flagella and post-acrosomal region of freshly collected caput epididymal sperm and cauda epididymal sperm (Figure 8A and B). A similar staining pattern was seen in cauda epididymal sperm that had been incubated in non-capacitating conditions (Figure 8E), capacitating conditions (Figure 8C), or physiological conditions in medium mimicking epididymal luminal fluid (Figure 8D). Similar to MFN2, MNS1 was present on the sperm flagellum but, in contrast to MFN2, MNS1 did not change its immunofluorescence pattern after incubation in standard media.

### MFN2 and MNS1 co-localize to the sperm flagellum

**Figure 9:**
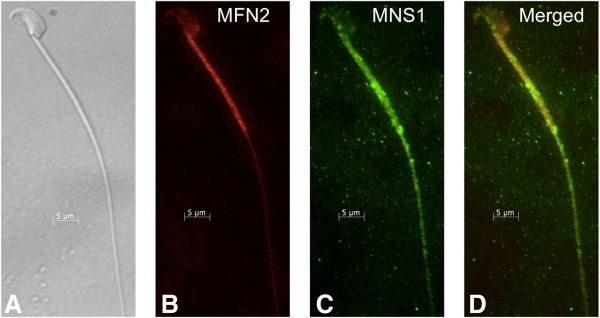
**Dual labeling immunofluorescence with anti-MFN2 and anti-MNS1 of cauda epididymal sperm to indicate co-localization.** Nomarski differential interference contrast micrograph of cauda epididymal sperm **(A)**. Anti-MFN2 fluorescent image of cauda epididymal sperm **(B)**. Anti-MNS1 fluorescent image of cauda epididymal sperm **(C)**. Merged image of cauda epididymal sperm probed with anti-MFN2 and anti-MNS1 **(D)**. Both proteins localized to the flagellar midpiece while MNS1 was also present throughout the entire flagellum. A 5 μm bar is indicated on all images.

Using indirect immunofluorescence, dual staining of cauda epididymal sperm with anti-MFN2 and anti-MNS1 was performed to determine the co-localization of MFN2 and MNS1. Anti-rabbit Alexa Fluor 568-conjugated secondary antibody was used to recognize anti-MFN2, and anti-mouse Alexa Fluor 488-conjugated secondary antibody was used to recognize MNS1. MFN2 localized to the midpiece of the flagellum (Figure 9B) while MNS1 was present as punctate staining throughout the length of the flagella of sperm (Figure 9C). Both proteins were observed in the post-acrosomal region of the sperm head. The merged image of anti-MFN2 and anti-MNS1 demonstrate co-localization of these two proteins in the post-acrosomal region and flagellar midpiece of cauda epididymal sperm (Figure 9D).

### MFN2 localizes to the mitochondria of the spermatogenic cell cytoplasm

**Figure 10:**
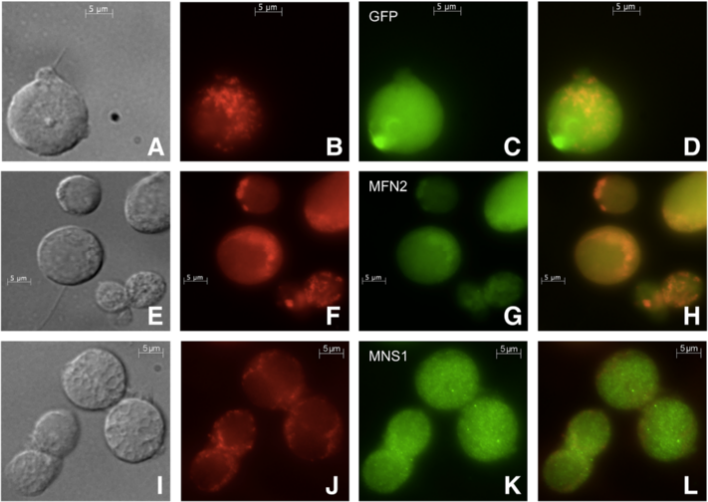
**Use of Red-Green transgenic mice to correlate antibody staining of MFN2 and MNS1 with mitochondria.** Panels **(A-D)** are unstained, fixed germ cells. RFP is expressed in the mitochondria **(B)**, and GFP is expressed in proacrosomal granules or the developing acrosomes **(C)**. Immunofluorescence of spermatogenic cells with anti-MFN2 **(E-H)** or anti-MNS1 **(I-L)** was performed. MFN2 localized to the germ cell mitochondria **(G)**. MNS1 was seen in a speckle pattern throughout the sperm cytoplasm **(K)**. Panels **(A, E, I)** are Nomarski differential interference contrast micrographs. Panels **(B, F, J)** display RFP in the mitochondria. Panels **(D, H, L)** are merged images. The second antibody is labeled with Alexa Fluor 488 (green) and are indicated in panels **(C, G, K)**. A 5 μm bar is indicated on all images.

Indirect immunofluorescence was used to examine the localization of MFN2 and MNS1 in developing spermatogenic cells of Red-Green transgenic mice that express RFP in their mitochondria and GFP in their sperm acrosomes [11]. Unstained, fixed spermatocytes displayed RFP in the mitochondria, which were located at the periphery of the cell (Figure 10B). GFP staining was detected in a localized area of proacrosomal granules [22] (Figure 10C). The merged image of unstained, fixed spermatogenic cells showed the distinct fluorescence pattern of RFP and GFP in these cells (Figure 10D). MFN2 localized to the mitochondria (Figure 10G). The localization of MFN2 to the spermatogenic cell mitochondria was seen in the merged imaged (Figure 10H) of RFP fluorescence (Figure 10F) and anti-MFN2 staining (Figure 10G). MNS1 was detected as punctate staining throughout the spermatogenic cell cytoplasm (Figure 10K). The merged imagine displayed the mitochondria around the periphery of the cell and MNS1 punctate staining throughout the cell cytoplasm (Figure 10L).

### MFN2 is present in the tail structures of cauda sperm

**Figure 11:**
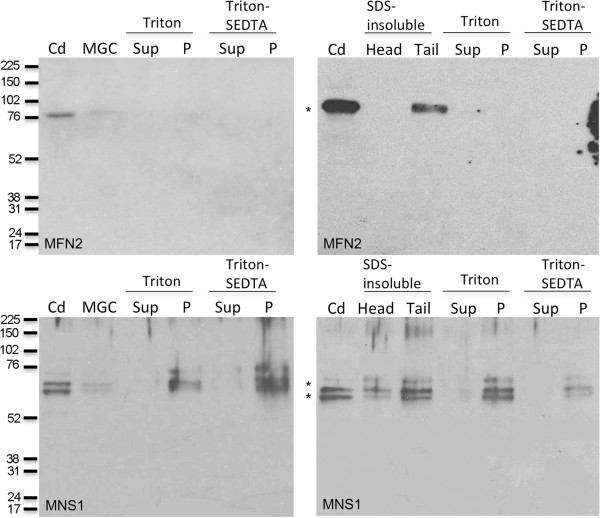
**SDS-PAGE and immunoblot of detergent extracted spermatogenic and sperm cells probed against anti-MFN2 and anti-MNS1.** Immunoblot with anti-MFN2 (top) or anti-MNS1 (bottom) antibodies to analyze (left panels): spermatogenic cells, Triton X-100 soluble and insoluble spermatogenic cell proteins, and Triton X-100/S-EDTA soluble and insoluble spermatogenic cell proteins; and (right panels): cauda epididymal sperm, SDS-insoluble head and tail proteins, Triton X-100 soluble and insoluble whole sperm proteins, and Triton X-100/S-EDTA soluble and insoluble whole sperm proteins. MFN2 was present in cauda epididymal sperm and in the tail structures of the whole sperm. MNS1 was present as closely migrating bands in cauda epididymal sperm and spermatogenic cells. MNS1 was also present in detergent-resistant structures of the sperm and spermatogenic cells. It was seen more abundantly in the tail structures of the whole sperm versus the head. Cd: cauda epididymal sperm, MGC: mixed germ cells, Sup: supernatant, P: pellet. Numbers on the left of the panels indicate the molecular weights of standard proteins (×10^-3^). Asterisk (*) indicates expected size (MFN2: *M*_*r*_ of 80 and MNS1: *M*_*r*_ of 60) of protein.

Spermatogenic cells and cauda epididymal sperm were extracted sequentially with a series of buffers containing detergents. The extracted proteins were subjected to SDS-PAGE and immunoblotting, and then probed with the antibodies against MFN2 and MNS1. SDS-treatment solubilizes the tubulin components of the axoneme [23]. The remaining axonemal components, ODFs, mitochondrial sheath, and fibrous sheath persist as the SDS-resistant structures. Although MFN2 was present in the mixed germ cell population and cauda epididymal sperm, it was not seen in the Triton X-100 or Triton X-100/S-EDTA supernatants extracted from the spermatogenic cells or cauda epididymal sperm. MFN2 was present in the SDS-resistant tail structures and was not observed in the SDS-resistant heads (Figure 11: Top).

In sperm and spermatogenic cells, MNS1 was present as three closely migrating bands (*M*_*r*_ of 72,000, 64,000, and 60,000) in structures resistant to detergent (Triton X-100, S-EDTA, Triton X-100/S-EDTA) extraction (Figure 11: Bottom). It was seen more abundantly in the SDS-resistant tail structures of the cauda epididymal sperm compared to SDS-resistant head structures, which may represent a slight contamination of the head structures with tail components. MNS1 existed as two closely migrating bands (*M*_*r*_ of 64,000 and 60,000), with a potential, faint, higher third band (*M*_*r*_ of 72,000) in the cauda epididymal sperm and spermatogenic cell samples. Similar to the results obtained above, these experiments demonstrated that MFN2 and MNS1 were associated with the sperm flagellar structures lacking the axoneme. Furthermore, the complete solubilization of both proteins required the utilization of reducing agent.

## Discussion

Here we hypothesize a model whereby MNS1 acts as a scaffolding protein in the sperm flagellum, interacting with the mitochondrial sheath and the fibrous sheath through communications with MFN2. This model is based on the homology between MNS1 and mitostatin (Figure 2). Recent discoveries have linked the latter protein with mitochondrial movement in somatic cells [6]. Mitostatin is associated with the mitochondrial outer membrane, and over-expression leads to reduced mitochondrial motility, whereas lack of it enhances mitochondrial movement. The activity appears to be mediated through binding the mitochondria to the actin intermediate filaments. In addition, mitostatin is involved in regulating mitochondrial-ER binding via MFN2 [6].

Further support for a role of mammalian MFN2 in mitochondrial dynamics in spermatogenic cells comes from studies in the fruit fly. Following meiosis in *Drosophila melanogaster* spermatogenesis, the spermatid mitochondria collect on one side of the haploid nucleus and fuse together into two giant aggregates; these two mitochondrial aggregates then wrap around each other to form a spherical structure termed the Nebenkern [24]. This process is governed by the fuzzy onions 1 (Fzo) protein, a large transmembrane GTPase associated with the outer mitochondrial membrane. Fzo is required for the formation of the giant mitochondrial derivatives during spermatogenesis, and loss of its function causes alterations of spermatogenesis and male sterility in *Drosophila*[24]. This protein is homologous to MFN1 and MFN2, providing important clues to their roles in mitochondrial physiology in mammals [25].

MFN1 and MFN2 sequences are highly homologous (81%) and display similar topology in the membrane of mitochondria [26], yet MFN2 appears to modulate more than mitochondrial fusion in mammalian cells. MFN1 and MFN2 possess an N-terminal GTPase domain and a coiled-coil domain located near the N-terminus that is exposed to the cytosol. In somatic cells, they are composed of two transmembrane domains that span the outer mitochondrial membrane as well as two regions for protein-protein interaction. The C-terminus ends in a coiled-coil motif. MFN2 possesses an N-terminal RAS binding domain [25]. MFN1 has higher GTPase activity and induces fusion more efficiently than MFN2. It requires a protein on the inner mitochondrial membrane, OPA1, for this function [27,28]. The increased mitochondrial fusion activity of MFN1 over MFN2 lends to the hypothesis that MFN2 plays a more diverse role in the cell.

Deletion of *Mfn1* or *Mfn2* impairs embryonic development in the mouse [29]. During embryogenesis there is a switch from glycolytic to oxidative metabolism in cells, which is, in part, regulated by the *Mfn* genes. In wild-type mouse embryonic fibroblasts, mitochondria move back and forth along the long axis of the cell on radial tracks. In contrast, the tubular shaped mitochondria from *Mfn1*^-/-^ embryonic fibroblasts are fragmented and display disorganized movement. The mutant embryos die midgestation [29]. In *Mfn2*^-/-^ embryonic fibroblasts, tubular mitochondria display organized movement yet spherically shaped mitochondria display uncoordinated motion. MFN2 is also required for placentation. *Mfn2*^-/-^ mutant embryos have a disruption of the placental trophoblast giant cell layer, and they also die midgestation [29]. *Mfn1*^-/-^ mutant embryonic cells have less fusion activity than *Mfn2*^-/-^ embryonic cells further indicating that MFN2 may play multiple roles in the cell beyond mitochondrial fusion.

Here, we began our studies by characterizing the presence of MFN2 during spermatogenesis and its localization within mouse spermatogenic cells and epididymal sperm. We report that MFN2 and MNS1 are interacting partners of the sperm flagellum and are present during spermatogenesis. Studies of expression by quantitative RT-PCR enabled us to determine at what stages *Mfn2* and *Mns1* expression began and in what cell types and tissues these proteins were present. A gene that is more highly expressed in spermatids relative to spermatocytes may be more likely to encode a protein that will function in flagellar biogenesis. Similarly, a protein that is highly expressed in ciliary tissues such as brain, trachea, kidney, and oviduct, may be involved in axonemal function. In *Drosophila*, Fzo protein is specifically and transiently expressed in spermatids [24].

Quantitative RT-PCR indicated that *Mfn2* and *Mns1* were highly expressed in spermatogenic cells decreasing from pachytene spermatocytes to condensing spermatids (Figure 4). Although this is not in line with an expected increased expression in spermatids *versus* spermatocytes, it is possible that these proteins are made in abundance during late meiosis to prepare the cell to begin flagellar biogenesis immediately following the second and final meiotic division. Nevertheless, the high level of expression in spermatids indicates a role for these proteins in flagellar biogenesis. While we do not see high expression of these genes in other tissues such as testis, brain, trachea, kidney, and oviduct by quantitative RT-PCR, we do see their protein products in these tissues. Testis, ovary, oviduct, and uterus samples displayed three additional MFN2 bands that could be variants specific to reproductive tissues. We found that the MNS1 protein existed as doublet bands in spermatogenic cells, epididymal sperm, and reproductive tissues, which may be a result of an alteration in the phosphorylation status of this protein. We also noted four additional bands in testis samples indicating potential testis-specific variants or protein degradation (Figure 6).

The use of co-immunoprecipitation provided evidence to support our hypothesis that MFN2 and MNS1 form a protein complex in spermatogenic cells. When MFN2 was used as bait, MNS1 was co-immunoprecipitated as two closely migrating bands (Figure 5). The reciprocal experiment was also true; when MNS1 was used as bait, MFN2 was co-immunoprecipitated (Figure 5). These experiments provide preliminary data for an interaction between these two proteins in developing spermatids. Further experiments are underway to investigate additional proteins that may be associated with the MFN2-MNS1 protein complex found in spermatogenic cells. We hypothesize that ODF2 is also a member of this complex.

Co-localization of MFN2 and MNS1 was observed in the sperm flagellum (Figure 9). This result lends further support to our hypothesis. The localization in epididymal sperm of each protein independently was determined to be flagellar with MFN2 being confined to the midpiece of the tail of freshly isolated sperm (Figure 7) and MNS1 being present throughout the sperm flagellum (Figure 8). However, when cauda epididymal sperm were incubated in non-physiological conditions, the pattern of MFN2 fluorescence was altered from being present in the flagellar midpiece to being present throughout the flagellum. This modification was not observed when sperm were maintained in a physiologic environment. The pattern of MNS1 fluorescence was not different, regardless of the conditions used to maintain the sperm. The alterations observed in MFN2 have been noted in boar sperm; however, these experiments did not investigate sperm maintained under conditions mimicking the lumen of the epididymis [8]. Our results indicate there could be unmasking of antigenic epitopes in the more distal regions of the sperm flagellum. The location of MFN2 in the sperm midpiece is logical because this is the location of all of the sperm mitochondria. However, the role of MFN2 in the post-acrosomal region and principal piece is unclear. We speculate a role for MFN2 in flagellar biogenesis and disassembly at fertilization.

To assess the biochemical nature of MFN2 and MNS1 localization in spermatogenic and sperm cells, we extracted these cells with a series of detergents. SDS-treatment solubilizes the tubulin components of the axoneme while other flagellar components such as the ODFs, mitochondrial sheath, and fibrous sheath remain in the SDS-resistant fraction [23]. MFN2 was observed in the SDS-resistant tail fraction of the sperm; however, it was not seen in the detergent-soluble fractions. Similarly, MFN2 was not present in the detergent-soluble fractions of the spermatogenic cell population. These results may be due to a low level of MFN2 protein in these extracts or, possibly, the epitope(s) recognized by the antibody is(are) hidden until it(they) is(are) exposed by SDS extraction. MNS1 was located in the detergent-resistant structures of spermatogenic cells and cauda epididymal sperm and was detected as three closely migrating bands by SDS-PAGE. It is possible that MNS1 exists in several forms in spermatogenic and sperm cells.

## Conclusions

The biogenesis of the sperm tail is an orderly process where each round spermatid elaborates a flagellum initially consisting of the axoneme surrounded by the plasma membrane. Subsequently, the flagellar accessory structures (mitochondrial sheath, ODFs, and fibrous sheath) are produced in a defined manner [30]. Several lines of evidence support that MFN2 and MNS1 are present and may play a role in flagellar and, potentially, ciliary biogenesis. First, *Mfn2* and *Mns1* expression occurred at high levels during spermatogenesis and their protein products are present. Second, we observed that both proteins were present in spermatogenic cells, caput and cauda epididymal sperm, and male as well as female reproductive tissues. Lastly, both proteins form a protein complex and co-localized to the sperm flagellum. In this manuscript we hypothesize a role for MNS1 whereby it acts as a scaffolding protein in the sperm flagellum, interacting with MFN2. Future studies are underway to substantiate this hypothesis further.

## Abbreviations

BLAST: Basic Local Alignment Search Tool; bp: Base pair; Br: Brain; BSA: Bovine Serum Albumin; C: Celsius; CaCl2: Calcium Chloride; Cd: Cauda; Cp: Caput; CS: Condensing Spermatids; Ct: Threshold Cycle; C-terminus: Carboxyl Terminus; DNA: Deoxyribonucleic Acid; DTT: Dithiothreitol; EDTA: Ethylenediaminetetraacetic Acid; EGTA: Ethylene Glycol Tetraacetic Acid; ER: Endoplasmic Reticulum; Fzo: Fuzzy Onions 1; g: Gravity; GAPDH: Glyceraldehyde 3-Phosphate Dehydrogenase; GFP: Green Fluorescent Protein; h: Hour; HCL: Hydrogen Chloride; H2O: Water; Hr: Heart; IgG: Immunoglobulin G; IP: Immunoprecipitation; KCL: Potassium Chloride; Kd: Kidney; kg: Kilogram; KH2PO4: Monopotassium Phosphate; Ln: Lung; Lv: Liver; M: Molar; MFN2: Mitofusin 2; MGC: Mixed Germ Cells; MgCl2: Magnesium Chloride; MgSO4: Magnesium Sulfate; min: Minute; ml: Milliliter; mM: Millimolar; MMW: Modified Whitten Medium; MNS1: Meiosis-Specific Nuclear Structural 1; mOsmol: Milliosmol; Mr: Molecular Mass; mRNA: Messenger Ribonucleic Acid; MW: Whitten Medium; NaCl: Sodium Chloride; NaHCO3: Sodium Bicarbonate; NaH2PO4: Monosodium Phosphate; Na2HPO4: Disodium Phosphate; NIH: National Institute of Health; N-terminus: Amino Terminus; Od: Oviduct; ODFs: Outer Dense Fibers; ODF2: Outer Dense Protein 2; OPA1: Optic Atrophy 1; Ov: Ovary; P: Pellet; PAGE: Polyacrylamide Gel Electrophoresis; PBS: Phosphate Buffered Saline; PCR: Polymerase Chain Reaction; PIPES: Piperazine-N,N’-Bis(2-Ethanesulfonic Acid); PS: Pachytene Spermatocytes; qPCR: Quantitative Polymerase Chain Reaction; RFP: Red Fluorescent Protein; RNA: Ribonucleic Acid; RS: Round Spermatids; RT-PCR: Reverse Transcription Polymerase Chain Reaction; SDS: Sodium Dodecyl Sulfate; S-EDTA: Sodium Dodecyl Sulfate Ethylenediaminetetraacetic Acid; Sk: Skeletal Muscle; Sp: Spleen; Sup: Supernatant; T: Testis; TBSS: Tris Buffered Saline High Salt; TBST: Tris Buffered Saline Tween; Tr: Trachea; TRIS: Tris (Hydroxymethyl) Aminomethane; Tween: Polysorbate 20; Ut: Uterus; μg: Microgram; μL: Microliter; μm: Micrometer.

## Competing interests

The authors declare that there is no conflict of interest that could be perceived as prejudicing the impartiality of the research reported.

## Authors’ contributions

MLV and GLG designed experiments. MLV and AML performed the experiments. MLV and GLG wrote the manuscript. All authors read and approved the final manuscript.
